# American Medical Association

**Published:** 1857-04

**Authors:** Robt. C. Foster

**Affiliations:** Secretary Amer. Med. Ass., Nashville, Tenn.


### *American Medical Association.*

The tenth meeting of the Association will be held at Nashville, on Tuesday, May the 5th, 1857.

All bodies entitled to representation in the Association, would very much further and facilitate its affairs by sending lists of their representatives at an early period to the undersigned.

ARTICLE SECOND OF THE CONSTITUTION.

"The members of this institution shall collectively represent and have cognizance of the medical profession in every part of the United States, and shall hold their appointment to membership either as delegates from local institions, as members by invitation, or as permanent members.

"*The Delegates* shall receive the appointment from permanently organized medical societies, medical colleges, hospitals, lunatic asylums, and other permanently organized medical institutions of good standing in the United States. Each delegate shall hold his appointment for one year, and until another is appointed to succeed him, and shall participate in all the business and affairs of the Association.

"Each local society shall have the privilege of sending one delegate to the Association, for every ten regular resident members, and one for every additional fraction of more than half this number.

"The faculty of every regularly constituted medical college, or chartered school of medicine, shall have the privilege of sending two delegates. The professional staff of every chartered or municipal hospital containing a hundred inmates or more, shall have the privilege of sending two delegates; and every other permanently organized medical institution of good standing shall have the privilege of sending one delegate.

"*The members by Invitation* shall consist of practitioners of reputable standing, from sections of the United States not otherwise represented at the meeting. They shall receive their appointment by invitation of the meeting, after an introduction from any of the members present, or from any of the absent permanent members. They shall hold their connection with the Association until the close of the annual session at which they are received, and shall be entitled to participate in all its affairs as in the case of delegates.

"*The Permanent Members* shall consist of all those who have served in the capacity of delegates, and of such other members as may receive the appointment by unanimous votes.

"Permanent members shall at all times be entitled to attend the meetings, and participate in the affairs of the Association, but without the right of voting; and when not in attendance, they shall be authorized to grant letters of introduction to reputable practitioners of medicine residing in their vicinity, who may wish to participate in the business of the meetings, as provided for members by invitation.

"Every member elect prior to the permanent organization of the annual meeting, or before voting on any question after the meeting has been organized, must sign these regulations, inscribing his name and address in full, specifying in what capacity he attends, and if a delegate, the title of the institution from which he has received his appointment."

Resolutions passed at the eighth meeting of the Association, held at Philadelphia:

*Resolved*, That no State or local society shall hereafter be entitled to representation in this Association that has not adopted its code of Ethics.

*Resolved*, That no State or local society that has intentionally violated or disregarded any article or clause in the code of Ethics, shall any longer be entitled to representation in this body.

*Resolved*, That no organization or institution entitled to representation in this Association, shall be considered in good standing, which has not adopted its code of Ethics."

Resolutions passed at the ninth meeting, held at Detroit:

"*Resolved*, That any new medical institution not heretofore represented in this body, be required to transmit to the Secretary, with the credentials of its delegates, evidence of its existence, capacity and good standing."

Medical presses throughout the Union are respectfully requested to copy the above resolutions at their earliest convenience.

ROBT. C. FOSTER,
Secretary Amer. Med. Ass., Nashville, Tenn.

